# Transcriptome Characterization and Identification of Molecular Markers (SNP, SSR, and Indels) in the Medicinal Plant *Sarcandra glabra spp*.

**DOI:** 10.1155/2021/9990910

**Published:** 2021-07-07

**Authors:** Yanqin Xu, Shuyun Tian, Renqing Li, Xiaofang Huang, Fengqin Li, Fei Ge, Wenzhen Huang, Yin Zhou

**Affiliations:** ^1^College of Pharmacy, Jiangxi University of Traditional Chinese Medicine, Nanchang 330004, China; ^2^Center of Applied Biotechnology, Wuhan University of Bioengineering, Wuhan 430415, China; ^3^College of Bioscience and Biotechnology, Wuhan University of Bioengineering, Wuhan 430415, China

## Abstract

*Sarcandra glabra* has significant metabolically active bioingredients of pharmaceutical importance. The deficiency of molecular markers for *S. glabra* is a hindrance in molecular breeding for genetic improvement. In this study, 57.756 million pair-end reads were generated by transcriptome sequencing in *S. glabra* (Thunb.) Nakai and its subspecies *S. glabra* ssp. *brachystachys*. A total of 141,954 unigenes with 646.63 bp average length were assembled. A total of 25,620 simple sequence repeats, 726,476 single nucleotide polymorphisms, and 42,939 insertions and deletions were identified, and the associated unigenes and differentially expressed genes were characterized. This work enhanced the molecular marker resources and will facilitate molecular breeding and gene mining in *S. glabra* spp.

## 1. Background

*Sarcandra glabra* (Chloranthaceae family) is an evergreen, smooth shrub, and grows up to one-meter height. It is originated in tropical climate of south Asia. It especially grows in China, Japan, Philippines, Vietnam, Korea, Cambodia, Malaysia, India, Sri Lanka, and other areas including areas of North, Central, and South America and some areas of the Pacific [1]. It has been known for its medicinal values in the treatment of joint and cancer problems [2]. *S. glabra* extract (SGE) contains high amount of powerful anti-inflammatory compounds including isofraxidin, terpenoid saponins, fumaric acid, caffeic acid, rosmarinic acid, and caffeoylquinic acid [2]. SGE is widely used in Traditional Chinese Medicines to help with arthritis, pain, swelling, and redness [3]. It may have multifarious medicinal values as anti-inflammatory [4], antitumor [5], anti-infection [1], and antioxidant [6] bioactivities as described in recent studies. SGE also has the ability to attenuate hyperglycemia [7], which could be useful to recycle the industrial wastes for beneficial by-products [2]. *S. glabra* seeds can be dried and roasted for human consumption. Besides, it can also be used as an in- or outdoor ornamental plant. Owing to its significant chemical, rheological, and pharmacological importance [1, 2], it is important to improve of *S. glabra* varieties with wider acceptance and enriched with bioactive components.

The marker-based breeding techniques have been widely adapted for plant improvement and genetic conservation [8]. The information about the availability of molecular markers for genetic mapping and map-based gene cloning is always a basic need for any breeding or genomic research. Among DNA markers, single nucleotide polymorphisms (SNPs), simple sequence repeats (SSRs), and insertions and deletions (InDels) are the most simple and valuable markers [9]. The microsatellites are tandem repeats of various mono-, di-, tri-, tetra-, penta-, and hexanucleotide sequence motifs of variable lengths [10], which have been identified and manipulated in various crops [11]. SSR markers have always been preferred for their user-friendly properties and for their random genome distribution, simplicity of use, high level of polymorphism, high clarity, low operational cost, reproducibility, hypervariability, ease of multiplexing, amenability to automation, and use with low quality DNA [11]. Recently, high-throughput sequencing methods have widely been adapted for rapid identification of SNP, SSR, and InDel markers, especially in nonmodel plants [12].

The next-generation sequencing of cDNA pool obtained from extracted RNA of plant tissues is generally known as RNA-seq. The RNA-seq or transcriptomics approach can be used to obtain a huge number of expressed sequences which can further be used to design molecular markers. RNA-seq-based SSRs, SNPs, and InDels are associated with protein coding genes and their relevant translated regions [13]. The transcriptome-based markers facilitate the understanding of their link with phenotypic variation and/or functional genes [14]. They are transferable among closely related species [13] because of their highly conserved domains [15]. Furthermore, transcriptomic SSRs may provide important information about evolution and conservational genetic variation of plant species [8, 11, 16]. Recently, different reports based on genomics [17], transcriptomics [18], metabolomics [18], and phenomics [19] have been reported in *S. glabra*. Various researches for characterization of extrachromosomal (chloroplast) genome [20–22] and to find the evolutionary relationship of various *S. glabra* subspecies [23] have also been presented. Furthermore, the amplified fragment-length polymorphism (AFLP) and microsatellite establishment [24, 25] and SNP marker manipulation studies [26] have also been carried out for molecular evaluation in *S. glabra*. Nonetheless, the identification and characterization of molecular markers are still lacking in *S. glabra* which greatly impair establishment of breeding programs.

In this study, we generated transcriptome sequences of *S. glabra* (Thunb.) Nakai and its subspecies *S. glabra* ssp. *brachystachys*. We mined the transcriptome data to identify and characterize the SSR, SNP, and InDel markers in order to enhance the genetic resources of this pharmacologically important plant. We also reported the differentially expressed genes and their functional annotations in both subspecies.

## 2. Results

### 2.1. Transcriptome Sequencing and De Novo Assembly

Two *S. glabra* species with contrasting morphological and physiological traits (Table 1) were selected for *de novo* transcriptome assembly. Using the Illumina hiseq xten platform, we generated 52.327 and 57.756 million raw reads for *S. glabra* (Thunb.) Nakai (SG) and the subspecies *S. glabra* ssp. *brachystachys* (CSH), respectively (Table 2). After cleaning the reads, 7,847.65 and 8,661.75 million bases with 46.03% and 45.77% GC contents (Additional Figure 1) and 92.72% and 92.64% *Q* > 30 were retained for CSH and SG, respectively (Table 2).

**Figure 1:**
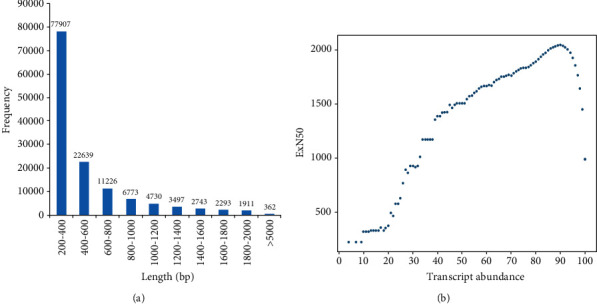
The clean reads after the sequence assembly: (a) length-based distribution of assembled reads and (b) distribution of N50 on the bases of expression clustering from 0-100.

The clean reads assembly provided 141,954 unigenes with an average length of 646.63 bp (Table 3). The majority unigenes (77,907) had short size (200-400 bp) while few unigenes (362) displayed very long size (>5,000 bp) (Figure 1(a)). The evaluation of expression quality (ExN50) of assembled contigs (N50) revealed that the majority of the contigs (>2,000 bp) showed the high expression quantity (86-92%) (Figure 1(b) and Additional Table 1).

The benchmarking universal single-copy orthologs (BUSCOs) were investigated, and 56.6%, 11.2%, and 15.4% of complete and single-copy, complete and duplicate-copy, and fragmented BUSCOs were observed, respectively (Table 3).

### 2.2. Transcript Annotation

The unigenes of SG and CSH after assembly were annotated using seven databases. Globally, 58,436 (41.17%) unigenes were annotated to at least one database. We successfully assigned and annotated GO terms to 28,948 (20.39%), 35,606 (25.08%), and 56,297 (39.66%) unigenes using the Pfam, UniProt, and NR databases, respectively (Table 3). Particularly, 34,857 unigenes (24.56%) revealed significant hits to the GO database. The GO terms classified into 147 groups and clustered in three classes and sixty subclasses (Additional Figure 2). Furthermore, 10.27% (14,575) unigenes were annotated to COG database (Additional Figure 3).

KEGG pathways analysis showed that 21,192 (14.93%) unigenes governed the 5 main and 33 subcategories of KEGG database (Additional Figure 4). The unigenes in “carbohydrate metabolism” were further evaluated for best functional orthologous group. In total, 187 unigenes were found for glutathione S-transferase (GST) as the top functional ortholog.

The species comparison analysis revealed 41.38% similarity with 10 species. Among the similarity hits, majority (37.18%) of the species showed 80-95% similarity. *S. glabra* had the highest transcript similarity with *Cinnamomum micranthum* f. kanehirae (9.71%) and *Nelumbo nucifera* (lotus root) (8.84%) (Additional Figure 5). The analysis of similarity distribution indicated the 37.18% sequences had 40-60% similarity, and 19.37% sequences had 80-95% similarity, while the 10.82% of sequences had 95-100% similarity (Additional Figure 6).

### 2.3. Expression and Pathway Enrichment Analyses

**Figure 2:**
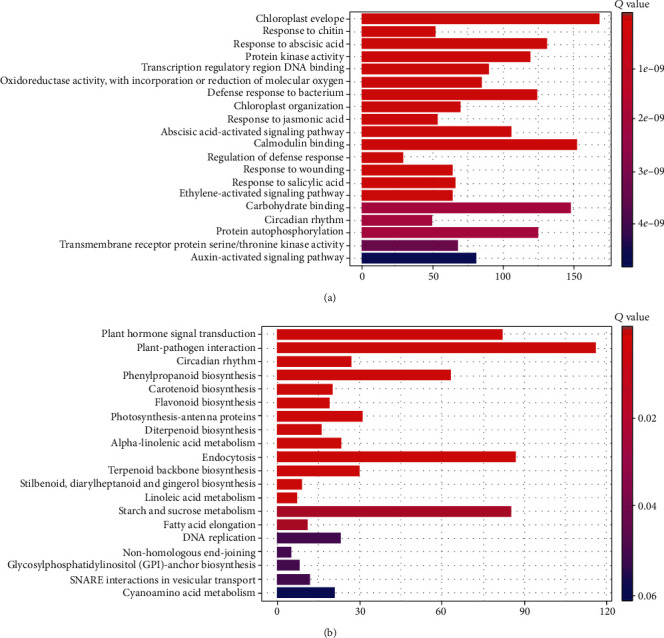
Classification of differentially expressed gene for functional annotations: (a) on the basis of Gene Ontology database (GO) and (b) through pathway significant enrichment database Kyoto Encyclopedia of Genes and Genomes (KEGG).

The nonstandard normal distribution was observed by transcript expression level based on FPKM values and density-distribution pattern for each sample (Additional Figure 7A). Transcripts with similar expression levels were clustered together (Additional Figure 7C & 7D). Pearson correlation analysis of the samples showed two separate groups for each subspecies (Additional Figure 7D), strong genetic similarity among biological replicates (*r*^2^ ≥ 0.9), while high dissimilarities were observed between CHS and SG. Among the mapped-reads, 14,584 unigenes were differentially expressed by more than twofold between GS and CHS (Additional Figure 7B). The DEGs were enriched in “chloroplast envelope,” “calmodulin binding,” and “carbohydrate binding” biological pathways (Figure 2). Furthermore, KEGG pathway analysis of the DEGs revealed “plant-pathogen-interaction,” “endocytosis,” and “plant-hormone signal-transduction” as the top enriched pathways (Figure 2).

### 2.4. Identification and Characterization of SSRs in the Transcriptomes

Only the unigenes with FPKM ≥ 1 were employed for detecting and SSRs. Collectively, 52,317,712 clean reads were assembled to 141,954 contigs of an average contig length of 646.63 bp. A total of 35,423 microsatellites were recognized on 25,620 contigs (Table 4 and Additional Table 2). The SSR density in the transcriptome was 385.91 SSRs/Mb. Among the SSRs, mononucleotide microsatellites were the most abundant (47.77%) with 16,920 bp length, followed by di- (33.85%), tri- (16.59%), tetra- (1.1%), hexa- (0.45%), and pentanucleotide (0.25%) types. The mononucleotide motifs A (50%) and T (48%) were highly abundant repeats. Among the total, 1,968 dinucleotide motifs GA (17.63%) and AG (16.61%) were the most detected types, while only 10 GC and 5 CG motifs were available in the transcriptome data. Among the trinucleotide, ATG (6.77%) followed by TCT (6.25%), and GAA (6.03%) motif types were identified, while only 8 CCG and 9 GCG were observed.

An average microsatellite length of *S. glabra* was 23.7 bp. The repeat motif size significantly affects the length variation of microsatellites. Hexanucleotide motifs with an average length of 34.8 bp were the longest motif followed by penta- (27.72 bp), di- (24.27 bp), tetra- (21.92 bp), and trinucleotide (19.7 bp) motifs. The mononucleotides showed the shortest average motif length of 12.56 bp. A trinucleotide motif 87 bp length and 29-fold repetition was the longest microsatellite observed (Table 4 and Additional Table 2). Other than these motifs, a compound motif type denoted as “c” for which two types of motifs separated by a few nonrepeating nucleotides, and “c^∗^” motifs with two different types of repeats without any separation were observed also. The 9.08% (3218) and 0.25% (90) of total SSRs were “c” and “c^∗^” type compound motifs, respectively, and showed an average length of 76.67 bp and 47.1 bp. Among the compound motifs, the longest was of 481 bp with 4 mononucleotide and 4 hexanucleotide motifs with multiple repeats. In the top ortholog group of carbohydrate metabolic pathway (K00799), a total 14 SSRs were identified for 19 assembled unigenes.

The widest range was observed for mononucleotides (10-80) in repetitions, while this range for di-, tri-, tetra-, penta-, and hexanucleotide microsatellites was 6 to 42, 5 to 29, 5 to 16, 5 to 8, and 3 to 11, respectively. Most of the motifs (5,749) had 10 repeats followed by 11 repeat motifs (3,854). The majority of unigenes (81%, 20,756) possessed only one microsatellite motif, while 15% (4,036), 2.68% (687), 0.46% (119), 17, and 4 unigenes had 2, 3, 4, 5, and 6 SSR motifs. Only one of the unigenes observed had nine SSR motifs.

### 2.5. SNPs and InDel Variant Identification, Distribution, and Their Selection Signature

We detected in the unigenes a total of 726,476 single nucleotide polymorphism sites (SNP) with an average of one SNP in 92.32 bp. These SNP markers were situated in 65,539 unigenes. The total number of transition and transversion mutations was 1,588,169 (63%) and 928967 (37%) (Table 5 and Additional Table 3). A total of 42,939 insertion and deletion (InDels) were recorded in the *S. glabra* assembled transcriptome. Out of them, 18,607 (43%) were insertions while 24,332 (57%) were deletion mutations. On an average, one InDel per 746 bp was found in 19,153 unigenes (Table 5 and Additional Table 4). In total, 89 SNP markers for 89 unigenes were related to 9 GO terms including sulfur compound metabolic process, response to stress, signal transduction, molecular functions, oxidoreductase-activity, transferase-activity transferring-alkyl or -aryl (other than methyl) groups, cellular components, cytoplasm, and nucleus, in the top ortholog group (K00799) of carbohydrate metabolic pathway (Additional Table 5).

The genetic variant (SNPs and Indels) distribution and the effect on gene functions were investigated. Totally, 36.894% (275,669) variants were observed in exon regions while 20.726% (154,861) variants were in intergenic regions. The variant numbers in 5′UTR and 3′UTR were 19.327% (144,409) and 23.051% (172,232), respectively. Only 22 variants were found in the splice site regions. The majority of variants (60.33%) have modifying effect in genome, which consisted of 3′UTR, 5′UTR, and intergenic variants. In contrast, 19.934% and 19% variants could exert low and moderate effects, respectively (Table 5). The missense variants were observed to be moderate effect variants. The start- and stop-codon-loss-in-function and stop-codon-gain-in-function variants were observed to have the high effect in the genome. The variants within the coding area mostly missense (52.116%) or silent (46.482%) while only 1.42% mutations were nonsense variations.

### 2.6. Validation of SSR Markers

Most of the two ends of SSRs are conservative single-copy sequences. The primers were designed as per complementary sequences of the two ends of the SSRs. A total of sixteen SSR markers including two markers for each type of SSRs were randomly selected for validation. Fifteen SSR markers were successfully amplified in 26 to 30 tested genotypes, while one marker was validated in 19 genotypes. The length polymorphism of SSR sites was displayed by electrophoresis of PCR products. One to 17 alleles could be identified. The description of validated markers, their PCR products, and polymorphic alleles are presented in Additional Table 6.

## 3. Discussion

The genetic improvement of *S. glabra* as a pharmacologically important plant is required, which could be accelerated by breeding strategies employing molecular markers. Two SG species (*S. glabra* ssp. brachystachys and *S. glabra* (Thunb.) Nakai) have various morphological differences including plant height, leaf shape, and appearance of inflorescences between them [27], and both are considered as model in previous studies for this family [28]. A molecular plant breeding program is always a fast and cost-effective tool in genomic improvement but it demands a rich molecular marker resource that is deficient for *S. glabra*. Development of such markers is not easy as its genome has not been sequenced yet. The transcriptome-based markers characterized in the study can be used to identify the linked and/or cosegregating loci for linkage and association-based genome mapping of various traits of interest in *S. glabra* as reported in various other plant species [29].

High-throughput sequencing of transcriptome is a fast and cost-effective way to characterize the genes and identify polymorphic molecular markers in nonmodel plants [12]. In the current study, we obtained the transcriptome data of *S. glabra* and revealed the available unigenes by *de novo* assembly. The high N50 and ExN90 values revealed the excellent sequencing quality. The coverage of functional genes was expressed by the BUSCO percentage. The relatively low percentage of complete BUSCOs may indicate the possibility that all genes in this species were not captured. It is normal as only the leaf samples and a single genotype per species were used in this study. It is known that the gene content and expression are different across tissues and genotypes. Furthermore, the transcriptome sequencing reveals only expressed functional genes, while a lot of genes present in the genome are missing. On the basis of transcriptome assembly, we characterized the molecular (microsatellite, SNP, and InDel) markers. The results indicated the availability of microsatellites in about one-fifth of the transcripts (18.04%), which is quite higher than the estimation of 2-5% in other reported studies [30]. Besides SSRs, we also detected SNPs and Indels which are considered very valuable markers. Recently, the development, characterization, and utilization of these markers in model crops as rice [31] and nonmodel crops as guar potato [11, 32] have been reported. The density of InDel and SNP identification was higher than the other studies as 17.10 SNPs per Kb in *Cyamopsis tetragonoloba* L. Taub [2] and one SNP per 17.08 Kb in Guar [32]. Collectively, the molecular markers obtained in this study will be helpful for future genomics studies and markers assisted selection in *S. glabra*. The leaf samples were employed for transcriptome characterization in this study because leaves are the main source of bioactive ingredients in SG. Nonetheless, mixing different tissues for transcriptome analysis may be a better approach in order to identify more transcripts and potentially more molecular markers as described in previous studies [33, 34].

The identified molecular markers in this study have tagged functional genes governing important metabolic activities including the ion binding. Various reports have shown the roles of ion channels in cancer from two principal standpoints: examining how specific ion channels are involved in certain cancer-related cellular behaviors such as proliferation, apoptosis, migration, or angiogenesis or examining the specific expression and functional profiles of various channel characteristic of certain human cancers [35]. Among the annotated pathways, the carbohydrate metabolism was observed to be on top hit. The evaluation of functional ortholog of this pathway indicated 187 unigenes for the glutathione S-transferase (GST) functional ortholog (k00799 in Map-05200) known as important genes in medicinal plants [22]. The glutathione S-transferases belong to a supergene family of seven cytosolic enzymes that catalyze the conjugation of glutathione (GSH) to a variety of electrophiles including arene oxides, unsaturated carbonyls, organic halides, and other substrates [35]. In humans, polymorphism in GST genes has been associated with susceptibility to various diseases. Previous studies described the associations between GST mutants (GSTM1 null and GSTT1 null) and location of colorectal cancers in individuals with mutation 1 in the MLH1 mismatch repair gene in Finnish kindreds. It is proposed that the GST genes are among other detoxicating enzymes that act as modifying genes and affect the phenotype in monogenic colorectal cancer [35].

Even though the establishment of AFLP and microsatellite markers have been reported in recent studies [24], and important molecular marker resources of *S. glabra* have been generated in this study, it is still less sufficient as compared to the well-studied model plants. Further works based on long-read transcriptome sequencing as well as whole genome sequences are needed to accelerate the genetic improvement of this important crop.

## 4. Methods

### 4.1. Plant Materials, Growth Conditions, and RNA Extraction

**Figure 3:**
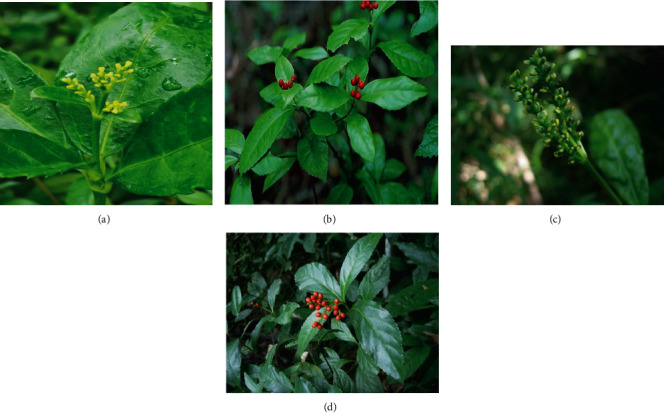
Morphological appearance of (a, c) inflorescence and (b, d) leaves of (a, b) *Sarcandra glabra* (Thunb.) Nakai and (c, d) subspecies *S. glabra* ssp*. brachystachys*.

The seeds of *Sarcandra glabra* (Thunb.) Nakai (SG) and its subspecies *S. glabra* ssp*. brachystachys* (Figure 3) were obtained from Hainan Branch Institute of Medicinal Plant Development, Chinese Academy of Medical Sciences, China. The selected, cleaned, and healthy seeds were grown in pots in a greenhouse with temperature 22-30°C and relative humidity 55-65%. The 30-day-old well-developed single plants were selected for RNA-seq study. Leaf tissues of SG and CSH were collected from three selected individual plants. The fresh tissues (100 mg) were used to extract the total RNA. The extracted RNA was further treated with DNase I for purification. The step by step process as total RNA sample detection, mRNA enrichment, double-strand cDNA-synthesis, end repair, splice selection and PCR-based amplification library quality detection, and computer sequencing was performed at Wuhan Benagen Tech Solutions Company Limited, Wuhan, China, to generate the paired-end reads.

### 4.2. Transcriptome Sequencing, Cleaning, and Assembly

The preparation of cDNA library and sequencing was performed by using three independent biological replicates for SG and CSH. The total RNA from each sample was extracted, and cDNA library was prepared. The original files of image data were obtained by high-throughput sequencing (Illumina hiseq xten) and were transformed into pair-end raw reads by base-calling analysis. As per machine's sequencing strategy, 150 bp average read length was maintained. The raw reads with joint sequences and/or <5 mass value, ≥50% proportion rate, more than or equal to 5% N base (the base with undetermined information), containing Poly-A, were filtered out to get the cleaned reads. The sequencing quality was estimated by *Q*Phred values. The clean reads were assembled into a fastq format [36]. The Trinity v 2.6.6 program [37] was used for transcriptome assembly and to get the unigenes with default parameters. The accuracy and effectiveness of the assembly results were ensured by estimation of N50, ExN50, and BUSCO [38].

### 4.3. Clustering and Functional Annotation

The extracted unigenes were manipulated by the Transcoder software v 4.1.0 to predict and translate the reading frames with default parameters. The blast X and blast P tools of diamond v 0.9.24 program [39] were used to compare the unigene sequences and protein sequences against UniProt database [40] with default parameters. At the same time, diamond v 0.8.36 program blast X was used to compare the unigene sequence with NR database to find the closely related species. Functional annotation was further performed by mapping unigenes to UniProt/Swiss port, Kyoto Encyclopedia of Genes and Genomes (KEGG) [41], EggNog [42], and Gene Ontology (GO) [43, 44] databases. The Hmmscan v3.1 (parameter: *E* value 0.01) was used for protein domain prediction on the basis of Pfam values.

### 4.4. Mining and Validation Analysis of SSRs

The MIcroSAtellite identification tool (MISA) v 1.0 was used to detect SSR loci from assembled contigs (http://pgrc.ipk-gatersleben.de/misa/misa.html). The minimum/standard repetition parameter was 10 for mono-, 6 for di-, and 5 for tri-, tetra, penta-, and hexanucleotide microsatellite motifs, respectively. The compound microsatellites were defined if the distance between two repeated motifs was shorter than 100 nucleotides [32]. Three primer pairs for all SSR markers were designed by primer 3 v2.3.5 [45] with default parameters. After SSR marker identification, sixteen SSR markers were randomly selected for further validation and a fixed sequence (GTAAAACGACGGCCAGT) was designed at the 5 ends of each of primer to match the fluorescent-labeled primer. PCR amplification with the designed primers of selected SSR markers was performed for DNA from fresh leaves of 31 genotypes including CSH4, CSH5, CSH6, CSH7, CSH8, SG31, SG32, SG33, SG34, SG42, SG44, SG45, SG48, SG49, SG50, SG53, SG55, SG2, SG4, SG5, SG13, SG14, SG15, SG16, SG18, SG19, SG20, SG23, SG28, and SG29 using DNeasy Plant Mini Kit (QIAGEN). All samples were obtained from College of Pharmacy, Jiangxi University of Traditional Chinese Medicine, China. After amplification, the fragment size was analyzed by Applied Biosystems 3737 sequencer.

### 4.5. Identification of SNPs and InDels

To identify the putative single nucleotide polymorphic sites (SNPs) and insertion and deletion events (InDels) in the transcripts, the high-quality transcriptome assembled sequences were aligned and compared by STAR v2.7.0 d program [46] with default parameters, and the BAM format comparison files were obtained. The Picard tool (version: 1.93) software package was employed to compare and process the results with default parameters. The SNP calling and low-quality filtering were performed by “haplotypecaller” in GATK (version: 4.1.4) [47] using default parameters. The SNP annotation was performed by “snpeff (version 4.3)” [48] using default parameters. Furthermore, the manual characterization of obtained SNP and InDel markers was performed.

### 4.6. Expression Analysis and Mining the Differentially Expressed Genes

The bowtie2 v 2.3.4 [49] with zero mismatch parameter was used to compare the reads to the assembled transcriptome. The results were compared with RSEM 2 (v 1.3.1) [50]. The number of read count on each gene was obtained from each sample, and the gene expression level was estimated by the FPKM method. The difference gene expression was analyzed by comparing read count data from the two subspecies using the DESeq2 program with the *Q*value <0.05 and log2FC > 1 as a threshold [35].

## Figures and Tables

**Table 1:** Comparison of key characteristics of *Sarcandra glabra* (Thunb.) Nakai and *S. glabra* subsp. *brachystachys* (Blume) Verdcourt.

Species	*S. glabra*	*S. glabra* subsp. *brachystachys*
Leaf	Leathery, margin sharply coarsely serrate except basally	Papery, margin dully serrate except basally
Stamen	Baculate to terete, thecae shorter than connective	Ovoid, thecae almost as long as the connective
Stigma	Subcapitate or minutely spotted	Minutely spotted
Fruit	Globose, shiny red or yellowish red at maturity	Ovoid, orange-red at maturity

**Table 2:** The summary for *Sarcandra glabra de novo* transcriptome assembly.

Sample name	^∗^CSH			^∗^SG		
	1	2	3	1	2	3
Raw reads						
Total raw reads	52,327,874	50,982,388	49,476,364	48,520,562	57,756,352	55,998,238
Total bases	7,849,181,100	7,647,358,200	7,421,454,600	7,278,084,300	8,663,452,800	8,399,735,700
GC content	46.03%	46.41%	45.76%	45.38%	45.76%	46.15%
Q20	97.62%	97.63%	97.60%	97.63%	97.61%	97.56%
Q30	92.72%	92.74%	92.66%	92.69%	92.64%	92.54%
Clean read						
Total reads	52,317,712	50,972,536	49,465,478	48,512,702	57,745,284	55,984,978
Total bases	7,847,656,800	7,645,880,400	7,419,821,700	7,276,905,300	8,661,792,600	8,397,746,700
GC content	46.03%	46.41%	45.76%	45.38%	45.77%	46.15%
Q20	97.63%	97.63%	97.60%	97.64%	97.61%	97.56%
Q30	92.72%	92.74%	92.67%	92.70%	92.64%	92.54%

^∗^CSH stands for *Sarcandra glabra* ssp. *brachystachys*, while SG represents *Sarcandra glabra* (Thunb.) Nakai.

**Table 3:** Characteristic descriptive and the functional annotation of *de novo* transcriptome assembly of *Sarcandra glabra.*

Descriptive	Value
Total length (bp)	91,791,960
Total unigene	141,954
GC contents (%)	41.91
N50 (bp)	989
N90 (bp)	264
Average (bp)	646.63
Median (bp)	363
Minimum (bp)	201
Maximum (bp)	17087
Contigs of size < 600 bp	77907
Contigs of size ≥ 600 bp	22669
Contigs of size ≥ 1000 bp	15536
Contigs of size ≥ 2,000 bp	362
^∗^Complete BUSCOs	291 (67.8%)
Complete and single-copy BUSCOs	243 (56.6%)
Complete and duplicated BUSCOs	48 (11.2%)
Fragmented BUSCOs	66 (15.4%)
Missing BUSCOs	72 (16.8%)
Total BUSCO groups searched	429 (100%)
Total annotations	58,436 (41.17%)
1: UniProt	35,606 (25.08%)
2: Pfam	28,948 (20.39%)
3: GO	34,857 (24.56%)
4: KEGG	21,192 (14.93%)
5: COG pathway	14,575 (10.27%)
6: EggNOG	23,086 (16.26%)
7: NR	56,297 (39.66%)

^∗^Complete BUSCOs: the detected gene length within the 95% confidence interval of the average length in the BUSCO homologous group, it may with single or multiple copies, while the incomplete BUSCOs are denoted as fragmented, and undetected BUSCO homologous group is denoted as missing.

**Table 4:** SSR motif repeat distribution in transcriptome data of *Sarcandra glabra.*

Number of repeats	SSR motifs						Compound motifs		Total
	Mono	Di	Tri	Tetra	Penta	Hexa	c	c^∗^	
5	0	0	2113	224	48	65			2450
6	0	1721	1004	62	8	41			2836
7	0	1085	559	6	7	15			1672
8	0	897	363	7	5	6			1278
9	0	665	99	1	0	1			766
10	5133	505	104	6	0	1			5,749
11	2717	1043	93	0	0	1			3,854
12	1808	267	67	0	0	0			2142
13	1096	116	63	1	0	0			1276
14	880	141	45	0	0	0			1066
15	687	138	31	0	0	0			856
16	541	142	35	1	0	0			719
17	390	138	11	0	0	0			539
18	266	170	4	0	0	0			440
19	217	157	6	0	0	0			380
20	173	170	2	0	0	0			345
>20	486	1,312	9	0	0	0			1807
Total	14394	8667	4608	308	68	130	3218	90	31483

**(a):** 

Type of SNP variants			Type of InDel variants		
Type	Count	Ratio	Type	Count	Ratio
3′UTR variant	172,232	23.05%	3′UTR variant	14,938	32.21%
5′UTR premature start codon gain variant	20,696	2.77%	5′UTR variant	12,609	27.19%
5′UTR variant	123,713	16.56%	Conservative in-frame deletion	1,195	2.58%
Initiator codon variant	94	0.01%	Conservative in-frame insertion	789	1.70%
Intergenic region	154,861	20.73%	Disruptive in-frame deletion	1,312	2.83%
Missense variant	142,027	19.01%	Disruptive in-frame insertion	871	1.88%
Splice region variant	25	0.00%	Frame-shift variant	6,919	14.92%
Start lost	520	0.07%	Intergenic region	5,782	12.47%
Stop gained	3,865	0.52%	Splice region variant	2	0.00%
Stop lost	1,027	0.14%	Start lost	471	1.02%
Stop retained variant	318	0.04%	Stop gained	237	0.51%
Synonymous variant	127,819	17.11%	Stop lost	1,257	2.71%

**(b):** 

Region wise			Region wise		
Exon	275,669	36.89%	Exon	11,086	24.96%
Intergenic	154,861	20.73%	Intergenic	5,782	13.02%
Splice sites	22	0.00%	Splice sites	1	0.00%
3′UTR	172,232	23.05%	3′UTR	14,938	33.63%
5′UTR	144,409	19.33%	5′UTR	12,609	28.39%

## Data Availability

The RNA-seq data has been submitted to NCBI SRA: PRJNA671629.

## Abbreviations

BUSCO: Benchmarking universal single-copy ortholog
CSH: *S. glabra* ssp. *brachystachys*
FPKM: Fragments per kilobase of transcript per million mapped reads
GBS: Genotyping-by-sequencing
InDels: Insertion and deletion events
SG: *S. glabra* (Thunb.) Nakai
SNP: Single nucleotide polymorphism
SSR: Simple sequence repeat.

## Supplementary Materials

Supplementary Materials Additional Figure 1: the base distribution of sample's reads. The first half is the base content distribution of the first end of the double end sequencing sequence, and the second half is the base content distribution of the other end. Additional Figure 2: simplified annotation information of Gene Ontology classification of identified transcripts for *S. glabra.* Additional Figure 3: the gene classification on the bases of similarity on cluster of indigenous groups of protein database for *S. glabra.* Additional Figure 4: the gene classification on the bases of KEGG metabolic pathways for *S. glabra.* Additional Figure 5: the comparison of *S. glabra* sequence with the other species, and the results for top 10 species have been presented while rests are classified into other species. Additional Figure 6: the transcriptome sequence evaluation for *S. glabra* by (A) comparison of evaluation values (*E* values) and (B) the sequence consistency analysis. Additional Figure 7: expression analysis: (A) The FPKM density map shows the overall gene expression pattern of each sample; (B) the MA plot displays the differentially expressed genes; (C) the expression patterns based on cluster analysis of all the DEGs in the different samples; (D) the correlation between samples based on DEGs. Additional Table 1: expression-based frequency distribution of transcripts extracted from transcriptome data of *S. glabra*. Additional Table 2: characterization of transcriptome SSR markers in *S. glabra.* Additional Table 3: characterization of transcriptome SNP markers in *S. glabra.* Additional Table 4: characterization of transcriptome InDel markers in *S. glabra.* Additional Table 5: the SSR, SNP, and InDel markers in transcriptome-based unigenes of *S. glabra* with the respective annotations in GO, KEGG, and COG databases. Additional Table 6: list of sixteen randomly selected and validated SSR markers, their primers, and PCR products.
