# Supplementary material for: Transcriptome Characterization and Identification of Molecular Markers (SNP, SSR, and Indels) in the Medicinal Plant Sarcandra glabra spp
Source: Biomed Res Int. 2021 Jul 7;2021:9990910. doi: 10.1155/2021/9990910 (PMC8282378; doi:10.1155/2021/9990910)
Supplement: Supplementary Materials — Additional Figure 1: the base distribution of sample's reads. The first half is the base content distribution of the first end of the double end sequencing sequence, and the second half is the base content distribution of the other end. Additional Figure 2: simplified annotation information of Gene Ontology classification of identified transcripts for S. glabra. Additional Figure 3: the gene classification on the bases of similarity on cluster of indigenous groups of protein database for S. glabra. Additional Figure 4: the gene classification on the bases of KEGG metabolic pathways for S. glabra. Additional Figure 5: the comparison of S. glabra sequence with the other species, and the results for top 10 species have been presented while rests are classified into other species. Additional Figure 6: the transcriptome sequence evaluation for S. glabra by (A) comparison of evaluation values (E values) and (B) the sequence consistency analysis. Additional Figure 7: expression analysis: (A) The FPKM density map shows the overall gene expression pattern of each sample; (B) the MA plot displays the differentially expressed genes; (C) the expression patterns based on cluster analysis of all the DEGs in the different samples; (D) the correlation between samples based on DEGs. Additional Table 1: expression-based frequency distribution of transcripts extracted from transcriptome data of S. glabra. Additional Table 2: characterization of transcriptome SSR markers in S. glabra. Additional Table 3: characterization of transcriptome SNP markers in S. glabra. Additional Table 4: characterization of transcriptome InDel markers in S. glabra. Additional Table 5: the SSR, SNP, and InDel markers in transcriptome-based unigenes of S. glabra with the respective annotations in GO, KEGG, and COG databases. Additional Table 6: list of sixteen randomly selected and validated SSR markers, their primers, and PCR products. [file 9990910.f1.zip › 9990910.f1/Additional Table 1.doc]

| Additional Table 1: Expression based frequency distribution of transcripts extracted from transcriptomic data of *Sarcandra glabra*. | | | | | | | | |
| --- | --- | --- | --- | --- | --- | --- | --- | --- |
| **Ex** | **min_Expr** | **ExN50** | **Transcripts_num** |  |  |  |  |  |
| E4 | 118182.391 | 221 | 1 |  |  |  |  |  |
| E7 | 99835.79 | 221 | 2 |  |  |  |  |  |
| E9 | 90835.091 | 221 | 3 |  |  |  |  |  |
| E10 | 50121.534 | 318 | 4 |  |  |  |  |  |
| E11 | 31077.781 | 318 | 5 |  |  |  |  |  |
| E12 | 31077.781 | 318 | 6 |  |  |  |  |  |
| E13 | 31077.781 | 329 | 7 |  |  |  |  |  |
| E14 | 31077.781 | 329 | 8 |  |  |  |  |  |
| E15 | 29879.279 | 329 | 9 |  |  |  |  |  |
| E16 | 27662.121 | 329 | 10 |  |  |  |  |  |
| E17 | 27662.121 | 354 | 11 |  |  |  |  |  |
| E18 | 18147.033 | 329 | 13 |  |  |  |  |  |
| E19 | 16370.677 | 355 | 15 |  |  |  |  |  |
| E20 | 16370.677 | 369 | 16 |  |  |  |  |  |
| E21 | 9910.778 | 492 | 19 |  |  |  |  |  |
| E22 | 9910.778 | 466 | 21 |  |  |  |  |  |
| E23 | 9910.778 | 575 | 24 |  |  |  |  |  |
| E24 | 9787.909 | 575 | 27 |  |  |  |  |  |
| E25 | 9266.139 | 628 | 31 |  |  |  |  |  |
| E26 | 5555.892 | 769 | 35 |  |  |  |  |  |
| E27 | 5555.892 | 892 | 40 |  |  |  |  |  |
| E28 | 4907.883 | 863 | 46 |  |  |  |  |  |
| E29 | 3909.621 | 925 | 53 |  |  |  |  |  |
| E30 | 3909.621 | 925 | 61 |  |  |  |  |  |
| E31 | 3387.794 | 915 | 70 |  |  |  |  |  |
| E32 | 2832.092 | 925 | 81 |  |  |  |  |  |
| E33 | 1984.384 | 1010 | 93 |  |  |  |  |  |
| E34 | 1744.269 | 1170 | 106 |  |  |  |  |  |
| E35 | 1621.994 | 1170 | 120 |  |  |  |  |  |
| E36 | 1516.297 | 1170 | 136 |  |  |  |  |  |
| E37 | 1186.906 | 1169 | 154 |  |  |  |  |  |
| E38 | 1186.906 | 1170 | 175 |  |  |  |  |  |
| E39 | 1093.439 | 1356 | 198 |  |  |  |  |  |
| E40 | 978.18 | 1386 | 223 |  |  |  |  |  |
| E41 | 892.095 | 1386 | 250 |  |  |  |  |  |
| E42 | 827.943 | 1420 | 279 |  |  |  |  |  |
| E43 | 815.627 | 1423 | 310 |  |  |  |  |  |
| E44 | 687.877 | 1423 | 345 |  |  |  |  |  |
| E45 | 574.792 | 1491 | 382 |  |  |  |  |  |
| E46 | 562.094 | 1463 | 423 |  |  |  |  |  |
| E47 | 499.871 | 1491 | 468 |  |  |  |  |  |
| E48 | 481.715 | 1505 | 516 |  |  |  |  |  |
| E49 | 435.479 | 1506 | 568 |  |  |  |  |  |
| E50 | 399.098 | 1505 | 625 |  |  |  |  |  |
| E51 | 398.203 | 1506 | 685 |  |  |  |  |  |
| E52 | 362.848 | 1546 | 749 |  |  |  |  |  |
| E53 | 311.738 | 1572 | 819 |  |  |  |  |  |
| E54 | 299.457 | 1576 | 893 |  |  |  |  |  |
| E55 | 273.376 | 1604 | 974 |  |  |  |  |  |
| E56 | 260.971 | 1617 | 1060 |  |  |  |  |  |
| E57 | 220.597 | 1640 | 1152 |  |  |  |  |  |
| E58 | 209.861 | 1658 | 1251 |  |  |  |  |  |
| E59 | 205.251 | 1666 | 1358 |  |  |  |  |  |
| E60 | 183.112 | 1666 | 1473 |  |  |  |  |  |
| E61 | 178.266 | 1673 | 1597 |  |  |  |  |  |
| E62 | 162.333 | 1668 | 1731 |  |  |  |  |  |
| E63 | 146.723 | 1702 | 1874 |  |  |  |  |  |
| E64 | 135.547 | 1722 | 2029 |  |  |  |  |  |
| E65 | 123.133 | 1730 | 2196 |  |  |  |  |  |
| E66 | 115.724 | 1750 | 2378 |  |  |  |  |  |
| E67 | 106.71 | 1750 | 2573 |  |  |  |  |  |
| E68 | 100.176 | 1761 | 2781 |  |  |  |  |  |
| E69 | 95.691 | 1768 | 3005 |  |  |  |  |  |
| E70 | 82.143 | 1761 | 3247 |  |  |  |  |  |
| E71 | 80.413 | 1782 | 3508 |  |  |  |  |  |
| E72 | 74.669 | 1801 | 3788 |  |  |  |  |  |
| E73 | 69.96 | 1815 | 4090 |  |  |  |  |  |
| E74 | 66.114 | 1830 | 4415 |  |  |  |  |  |
| E75 | 58.277 | 1834 | 4768 |  |  |  |  |  |
| E76 | 52.617 | 1835 | 5152 |  |  |  |  |  |
| E77 | 48.467 | 1842 | 5570 |  |  |  |  |  |
| E78 | 45.509 | 1858 | 6026 |  |  |  |  |  |
| E79 | 42.137 | 1876 | 6521 |  |  |  |  |  |
| E80 | 38.805 | 1890 | 7064 |  |  |  |  |  |
| E81 | 33.904 | 1911 | 7666 |  |  |  |  |  |
| E82 | 31.613 | 1934 | 8331 |  |  |  |  |  |
| E83 | 27.89 | 1956 | 9064 |  |  |  |  |  |
| E84 | 24.406 | 1974 | 9879 |  |  |  |  |  |
| E85 | 22.03 | 1995 | 10793 |  |  |  |  |  |
| E86 | 19.769 | 2013 | 11829 |  |  |  |  |  |
| E87 | 16.85 | 2023 | 13017 |  |  |  |  |  |
| E88 | 14.554 | 2032 | 14376 |  |  |  |  |  |
| E89 | 12.506 | 2039 | 15952 |  |  |  |  |  |
| E90 | 10.268 | 2044 | 17802 |  |  |  |  |  |
| E91 | 9.195 | 2038 | 19993 |  |  |  |  |  |
| E92 | 7.81 | 2023 | 22627 |  |  |  |  |  |
| E93 | 6.521 | 2004 | 25838 |  |  |  |  |  |
| E94 | 4.942 | 1974 | 29823 |  |  |  |  |  |
| E95 | 4 | 1925 | 34850 |  |  |  |  |  |
| E96 | 3.071 | 1856 | 41412 |  |  |  |  |  |
| E97 | 2.288 | 1766 | 50304 |  |  |  |  |  |
| E98 | 1.576 | 1642 | 62969 |  |  |  |  |  |
| E99 | 0.892 | 1448 | 83267 |  |  |  |  |  |
| E100 | 0 | 989 | 141954 |  |  |  |  |  |
